# REG4 Is Highly Expressed in Mucinous Ovarian Cancer: A Potential Novel Serum Biomarker

**DOI:** 10.1371/journal.pone.0151590

**Published:** 2016-03-16

**Authors:** Laura Lehtinen, Pia Vesterkvist, Pia Roering, Taina Korpela, Liisa Hattara, Katja Kaipio, John-Patrick Mpindi, Johanna Hynninen, Annika Auranen, Ben Davidson, Caj Haglund, Kristiina Iljin, Seija Grenman, Harri Siitari, Olli Carpen

**Affiliations:** 1 Department of Pathology, University of Turku and Turku University Hospital, Turku, Finland; 2 VTT Technical Research Centre of Finland, Espoo and Turku Centre for Biotechnology, University of Turku, Turku, Finland; 3 FIMM, Institute for Molecular Medicine Finland, University of Helsinki, Helsinki, Finland; 4 Department of Obstetrics and Gynecology, Turku University Hospital, University of Turku, Turku, Finland; 5 Department of Pathology, Oslo University Hospital, Norwegian Radium Hospital, Oslo, Norway; 6 University of Oslo, Faculty of Medicine, Institute of Clinical Medicine, Oslo, Norway; 7 Department of Surgery, University of Helsinki and Helsinki University Hospital, Helsinki, Finland; 8 Research Programs Unit, Translational Cancer Biology, University of Helsinki, Helsinki, Finland; The University of Texas MD Anderson Cancer Center, UNITED STATES

## Abstract

Preoperative diagnostics of ovarian neoplasms rely on ultrasound imaging and the serum biomarkers CA125 and HE4. However, these markers may be elevated in non-neoplastic conditions and may fail to identify most non-serous epithelial cancer subtypes. The objective of this study was to identify histotype-specific serum biomarkers for mucinous ovarian cancer. The candidate genes with mucinous histotype specific expression profile were identified from publicly available gene-expression databases and further in silico data mining was performed utilizing the MediSapiens database. Candidate biomarker validation was done using qRT-PCR, western blotting and immunohistochemical staining of tumor tissue microarrays. The expression level of the candidate gene in serum was compared to the serum CA125 and HE4 levels in a patient cohort of prospectively collected advanced ovarian cancer. Database searches identified REG4 as a potential biomarker with specificity for the mucinous ovarian cancer subtype. The specific expression within epithelial ovarian tumors was further confirmed by mRNA analysis. Immunohistochemical staining of ovarian tumor tissue arrays showed distinctive cytoplasmic expression pattern only in mucinous carcinomas and suggested differential expression between benign and malignant mucinous neoplasms. Finally, an ELISA based serum biomarker assay demonstrated increased expression only in patients with mucinous ovarian cancer. This study identifies REG4 as a potential serum biomarker for histotype-specific detection of mucinous ovarian cancer and suggests serum REG4 measurement as a non-invasive diagnostic tool for postoperative follow-up of patients with mucinous ovarian cancer.

## Introduction

Epithelial ovarian cancer (EOC) is the most lethal gynecological cancer [1]. Although new treatment modalities have prolonged survival during recent decades, the overall prognosis has not improved [2,3]. The symptoms, such as abdominal pain, increased abdominal size, bloating or nausea, are often non-specific until late in disease progression, leading to delayed diagnosis. Most patients are diagnosed at an advanced stage and treated by surgery combined with platinum-taxane chemotherapy. EOC consists of six main histological subtypes (histotypes); low-grade serous carcinoma (LGSC), high-grade serous carcinoma (HGSC), clear cell carcinoma (CC), endometrioid carcinoma (EC), mucinous carcinoma (MC) and transitional cell carcinoma (TC) with distinct genetic profiles, biological behavior and outcome.

The diagnosis of ovarian cancer relies on clinical features, ultrasound imaging and serum biomarker Cancer antigen 125 (CA125). In addition, the novel serum biomarker Human epididymis 4 (HE4) may bring additional information in differential diagnostics of women with pelvic mass [4]. These biomarkers, however, are not completely reliable for diagnosing non-serous histotypes. CA125 values have been shown to be lower for patients with MC compared to patients with SC [5,6]. Since the diagnosis cannot be based on the current serum biomarkers, in most cases the correct diagnosis is only revealed upon surgical intervention. Globally, mucinous carcinomas account for ~11% of EOC [7]. Although the prognosis is usually favorable, in some cases carcinomas recur and progress aggressively [8,9]. Furthermore, mucinous ovarian tumors have been shown to have lower response rate to platinum-based chemotherapy when compared to HGSC [5,6]. An increasing number of studies indicates that different ovarian cancer histotypes should be regarded as distinct diseases and therefore require different treatment [10]. Non-invasive diagnostic methods, such as histotype differentiating serum tests, could be valuable for optimization of treatment strategy before surgery.

This study aimed at identification of mucinous histotype specific serum biomarkers for ovarian cancer. Based on *in silico* analyses, we studied the expression of Regenerating Islet-Derived Protein IV (REG4) in a set of mucinous ovarian cancer tissues, patient sera and ascites fluids. In addition, REG4 concentration was measured in a time series of serum samples to determine the correlation to tumor burden. Furthermore, we examined CA125, HE4 and REG4 in the same samples to compare their performance.

## Materials and Methods

### *In silico* data mining

The MediSapiens database (www.medisapiens.com) was applied to study the gene expression levels across all normal and neoplastic human tissues [11]. The samples included in this database have been analyzed on the Affymetrix platform and, because of the unique normalization and data quality verifications, the gene expression profiles collected from different studies can be combined to generate an overview of the expression profile in human tissues. The data from normal ovary (n = 10) and ovarian cancer samples (n = 288) available in the MediSapiens database were utilized in tissue specific expression analysis.

### Clinical material

The studies involving clinical material have been performed in accordance with the ethical standards laid down in the 1975 Declaration of Helsinki. The ovarian tissue microarray (TMA) was constructed from surgical specimens collected from the tissue archive of Department of Pathology, Turku University Central Hospital as previously described [12]. The use of the specimens was approved by the The Ethics Committee of the Hospital District of Southwest Finland. Patient sera and part of the frozen tumor tissue specimens were collected from patients treated at Turku University Hospital. The serum samples included both treatment-naïve preoperative samples and longitudinal samples during treatment. The use of all clinical material was approved by The Ethics Committee of the Hospital District of Southwest Finland (ETMK, 53/180/2009 § 238) and The National Supervisory Authority for Welfare and Health (Valvira, DNRO 6550/05.01.00.06/2010 and STH507A). Additional fresh frozen mucinous ovarian tumor material was obtained from the Oslo University Hospital, Norway. Study approval was given by the Regional Committee for Medical Research Ethics in Norway and informed consent was signed by Norwegian patients.

### Tissue microarray and immunohistochemistry

The TMA contained 185 samples from 100 patients with ovarian neoplasms; serous cystadenomas (n = 16), serous borderline tumors (n = 27), serous carcinomas (n = 47), mucinous cystadenomas (n = 14), mucinous carcinomas (n = 36), endometroid carcinomas (n = 45). The TMA was stained with mouse monoclonal REG4 antibody [13], as described [14]. REG4 immunostaining was independently evaluated by two investigators (TK and OC); in case of disconcordance a consensus score was used for further evaluation.

### Quantitative RT-PCR

Total RNA was extracted from frozen ovarian tumor samples (10 mg piece) and primary cells using RNeasy Mini kit (Qiagen) according to the manufacturer’s protocol and processed to cDNA (Applied Biosystems, Foster City, CA). TaqMan probes were acquired from the Universal Probe Library (Roche Diagnostics, Espoo, Finland) and primers were ordered from Oligomer (Helsinki, Finland) for REG4 (forward: atgcggctgctcctattg; reverse: taaaaccatccaggagcaca) and GAPDH (forward: tggctctaccttagaaccctga; reverse: ttttgctctttgccgtacct). Real-time quantitative RT-PCR (qRT-PCR) was done with ABI Prism 7900 (Applied Biosystems). Quantitation was carried out with RQ manager 1.2 software using the _ΔΔ_CT method (Applied Biosystems). Three or more replicate samples were used to study the mRNA expression of REG4 and GAPDH. Average expression of the control samples was considered for the calculation of the fold changes and GAPDH was used as an endogenous control. Three or more replicate samples were studied for detection of mRNA expression. Statistical significance was determined with student’s t-test, and p-values < 0.05 were considered statistically significant.

### Western blotting

For protein lysates, approximately 20 mg pieces were cut from frozen tumors and lysed with sonication in RIPA lysis buffer. Protein concentrations were measured using BioRad protein assay kit according to manufacturer’s instructions (Bio-Rad Laboratories, CA). Samples were prepared mixing appropriate amounts of protein lysate with 5 x Laemmli buffer and boiled for 5 min. Equal amounts of protein (20 μg) were separated on 15% SDS-PAGE gel and transferred to nitrocellulose membrane (pore width 0,2 μm). Equal loading was confirmed with Ponceau staining. Membranes were blocked with 5% milk-TBST and probed with polyclonal REG4 antibody (1:1000, AF1379, R&D Systems, Minneapolis, MN) and HRP-conjugated anti-goat secondary antibody (1:5000, Dako, Glostrup, Denmark). Bound proteins were detected by enhanced chemiluminescence.

### ELISA assay

The REG4 sandwich ELISA assays were performed using the Human REG4 ELISA Pair Set (Sino Biological Inc., Beijing, China) according to the manufacturer’s instructions. The serum samples were diluted 1:1000 and ascites samples 1:500 in the sample dilution buffer. A pool of serum samples from healthy males (n = 4) was used as a negative control. The absorbance at 450 nm was measured with Victor 1420 Multilabel Counter (Perkin Elmer, Waltham, MA). All controls and samples were analyzed in two replicate wells in three individual runs.

## Results

### *In silico* data mining identifies REG4 as a biomarker candidate for mucinous ovarian cancer

In order to identify putative serum biomarker candidates for ovarian cancer detection and histotype specification, we performed *in silico* analysis of gene expression data using the GTI algorithm [15] and the MediSapiens database [11]. The analysis resulted in a list of 57 genes with mucinous histotype specific mRNA expression profile (S1 Table). The aim was to concentrate on genes highly expressed in malignant tissues with a secreted translational product. From the resulting candidates, already known ovarian cancer biomarkers were ruled out (e.g. SPINK1/TATI [16]), after which a more detailed analysis of the *in silico* expression data as well as relevant literature was conducted. The final candidate selection identified the Regenerating Islet-Derived Protein IV (REG4) as a potential serum biomarker for mucinous ovarian cancer histotype. REG4 is a member of the regenerating gene (REG) family, which belongs to the calcium-dependent lectin superfamily. It consists of 17 members, from which REG1, REG2, REG3 and REG4 have been associated with inflammation, diabetes and cancers [17].

**Fig 1:**
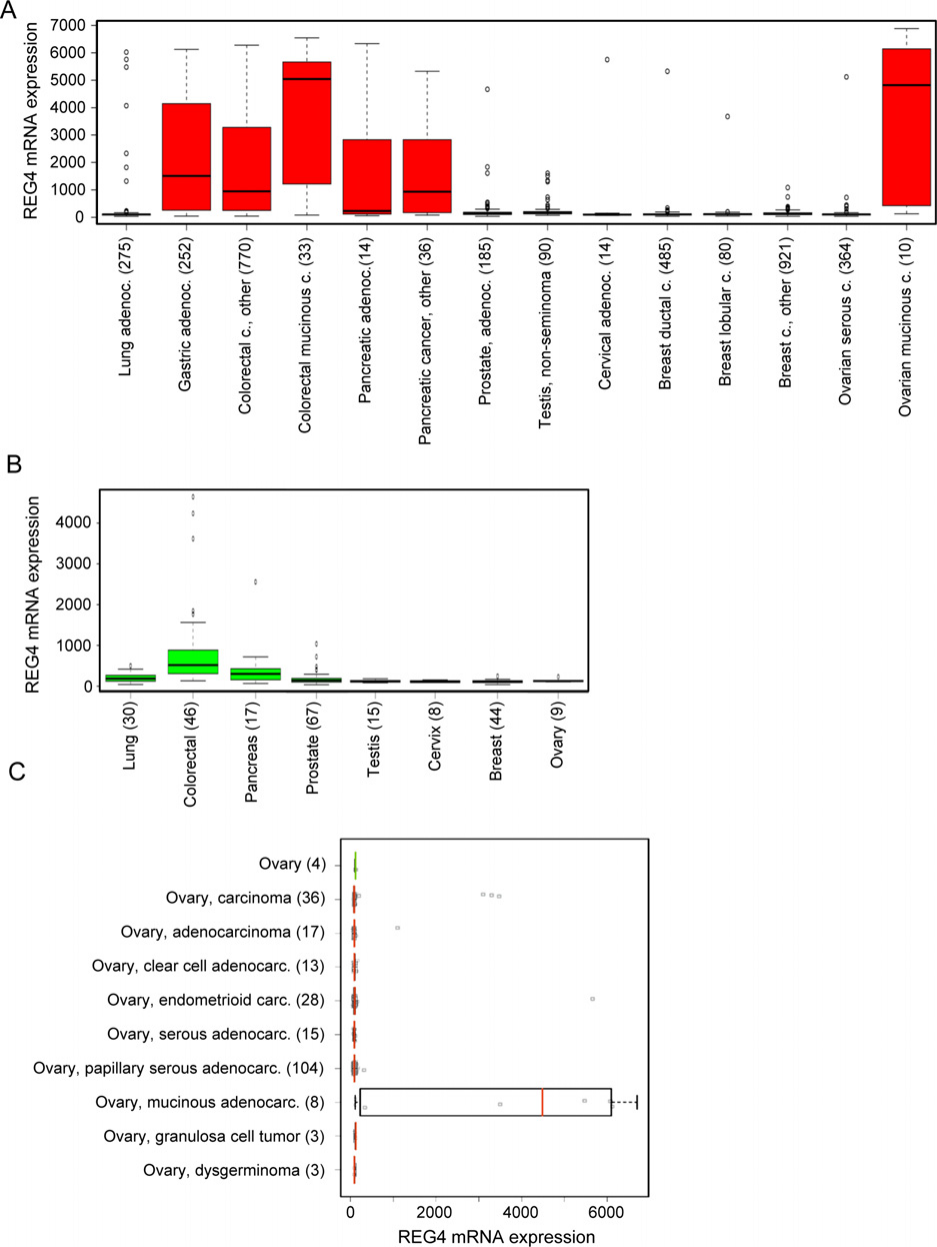
*In silico* data mining identifies REG4 as mucinous ovarian cancer specific serum biomarker candidate. (A) Malignant tissues with high REG4 mRNA expression (exp. value > 500, n = 14). (B) REG4 mRNA expression in corresponding healthy tissues (if available, n = 8). (C) Detailed analysis of REG4 mRNA expression in healthy and malignant ovarian tissues. The box refers to quartile distribution (25–75%) range, with the median shown as a vertical line (green in healthy samples, red in malignant). Data observations which lie more than 1.5*inter-quartile range higher than the third quartile, are considered as outliers and indicated separately.

*REG4* mRNA expression levels were studied in more detail using the MediSapiens database which includes expression data of human tissue samples (see S1 and S2 Figs for complete results). The overall *REG4* mRNA expression was significantly stronger in malignant tissues (n = 15392) than in healthy tissues (n = 3082). The results are summarized in Fig 1, showing malignant tissues (Fig 1A) with the highest REG4 mRNA expression levels (>500 in any sample) and their corresponding healthy tissues (Fig 1B). High expression of *REG4* mRNA was found in malignant gastric, colorectal and pancreatic tissues as well as in healthy colorectal and pancreatic tissues. The highest level of *REG4* mRNA expression was seen in mucinous ovarian cancer, while the expression level remained very low in healthy ovarian tissue. More specific analysis of *REG4* mRNA expression in healthy ovary (n = 10) and malignant (n = 288) ovarian tissues of various histologies, confirmed the mucinous histotype specific expression profile (p<0.001, Fig 1C). In other ovarian cancer histotypes, *REG4* mRNA expression remained at the level of healthy tissues.

### REG4 is highly expressed in tumor samples derived from mucinous ovarian cancer

**Fig 2:**
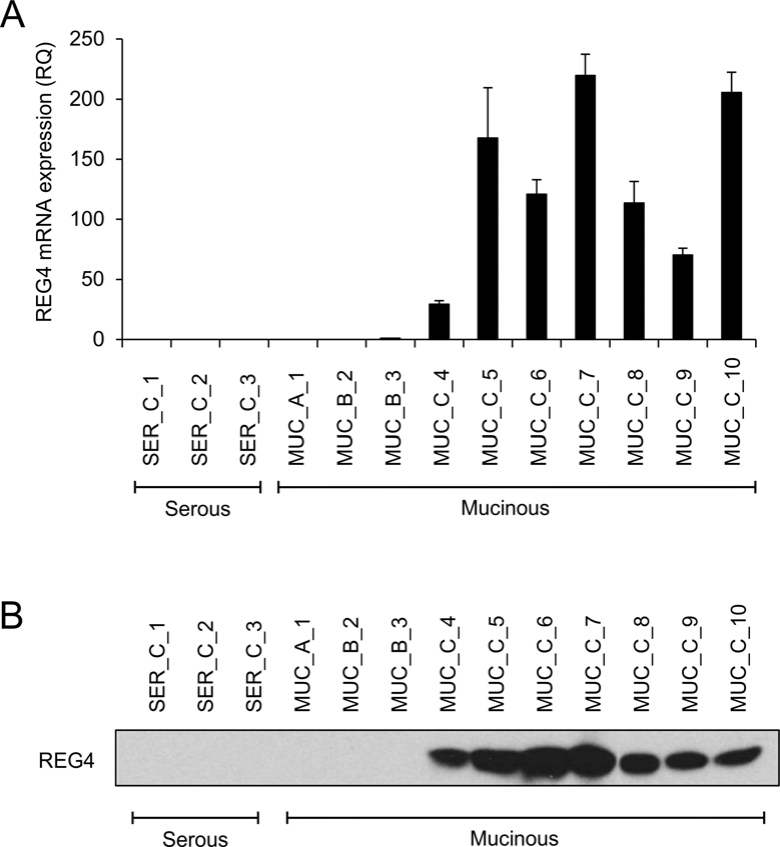
REG4 is highly expressed in tumor samples derived from mucinous ovarian cancer. (A) Quantitative RT-PCR analysis of REG4 mRNA expression in serous carcinomas (SER_C), mucinous cystadenomas (MUC_A), mucinous borderline tumors (MUC_B) and mucinous carcinomas (MUC_C). (B) Western blot analysis of REG4 protein expression in the same set of samples. The total protein content of each sample was 20 μg and equal loading was confirmed with ponceau staining.

The *in silico* analysis indicated specific expression of *REG4* mRNA in mucinous histotype of ovarian tumors. In order to validate these data, *REG4* mRNA and protein expression was studied in samples from mucinous (MUC) and high-grade serous (SER) ovarian tumors. The *REG4* mRNA expression was studied with qRT-PCR using mRNA from serous tumors as negative control. The results are summarized in Fig 2A. The REG4 mRNA showed high overall expression in mucinous carcinomas (MUC_C_4–10, Fig 2A) while no expression was detected in high-grade serous tumors (SER_1–3). Interestingly, *REG4* was not expressed in mucinous cystadenoma (MUC_A_1) and showed significantly lower mRNA expression levels also in mucinous borderline tumors (MUC_B_2–3). REG4 protein expression in the same set of tumor samples was assessed with western blotting. The results indicated high REG4 protein levels in *REG4* mRNA positive tumors (Fig 2B). Overall, REG4 protein expression was either very high or very low in these samples while mRNA analysis also identified samples with intermediate or low expression. These results validate the findings of the *in silico* analysis and indicate that REG4 expression is specific for mucinous ovarian cancer histotype.

### REG4 is expressed by goblet-like cells of malignant mucinous ovarian tumors

**Fig 3:**
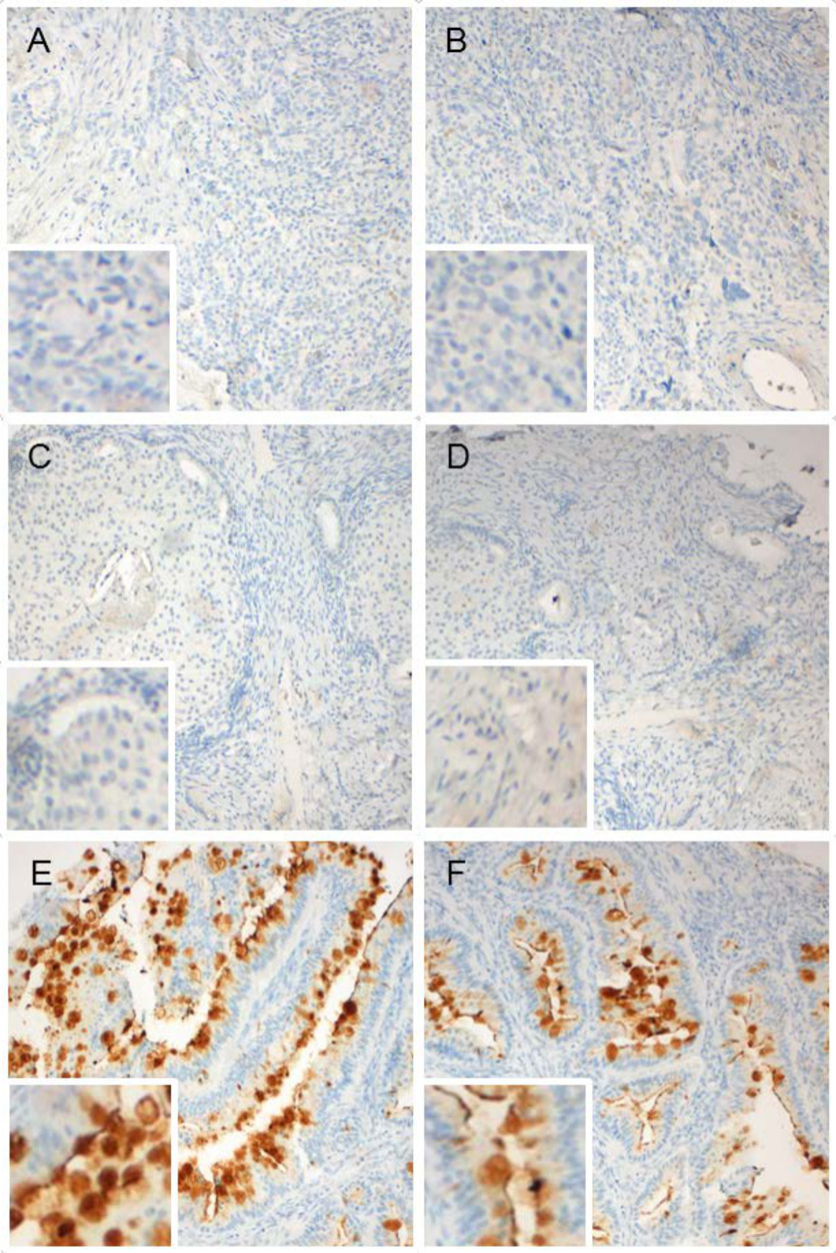
REG4 is expressed by goblet-like cells of malignant mucinous ovarian tumors. Immunohistochemical staining of REG4 in a TMA of malignant ovarian tissues. Examples of serous carcinoma (A-B), endometrioid carcinoma (C-D) and mucinous carcinoma (E-F) are presented.

REG4 expression pattern was studied in TMAs containing clinical samples from ovarian tumors with different histotypes. The results are summarized in Table 1 and in Fig 3. Concurring with the previous analyses, no staining was detected in serous (n = 90) or endometrioid neoplasms (n = 45), while positive REG4 staining was found in 83% (30/36) of mucinous carcinomas. This indicates that REG4 expression is specific for mucinous histotype. In all REG4 positive samples the staining was very strong. The mucinous tissue samples were confirmed to be of primary ovarian tumor origin by examining the longitudinal electronic health record information of each patient with special reference to previous diagnoses, diagnostic examinations and laparotomy status, as well as with CK7/CK20 staining. The TMA also contained samples of benign mucinous cystadenomas (n = 14). Importantly, no REG4 expression was detected in these samples. Correlation of staining results with patient data showed no association of REG4 expression to other than mucinous histotype. Strong REG4 staining was detected equally in low and high grade mucinous carcinomas. More detailed examination of the REG4 staining pattern revealed the strongest staining inside and between the mucinous inclusions of goblet-like tumor cells. Furthermore, REG4 was located exclusively on the apical surface of the tumor cells, while the stroma was negative.

**Table 1:** Immunohistochemical staining of REG4 in ovarian epithelial tumor tissues.

		REG4 staining		
Tumor type	No. of samples	pos	neg	Percentage of positive samples (%)
Serous carcinoma	47	0	47	0
Serous borderline	27	0	27	0
Serous cystadenoma	16	0	16	0
Endometrioid cystadenocarcinoma	45	0	45	0
Mucinous carcinoma	36	30	6	83
Grade 1	18	16	2	80
Grade 2	8	4	4	50
Grade 3	2*	2	0	100
Grade n/a	8	8	0	100
Mucinous cystadenoma	14	0	14	0

* Only one analyzed tumor

### Secreted REG4 can be detected from serum and ascites of mucinous cancer patients

**Fig 4:**
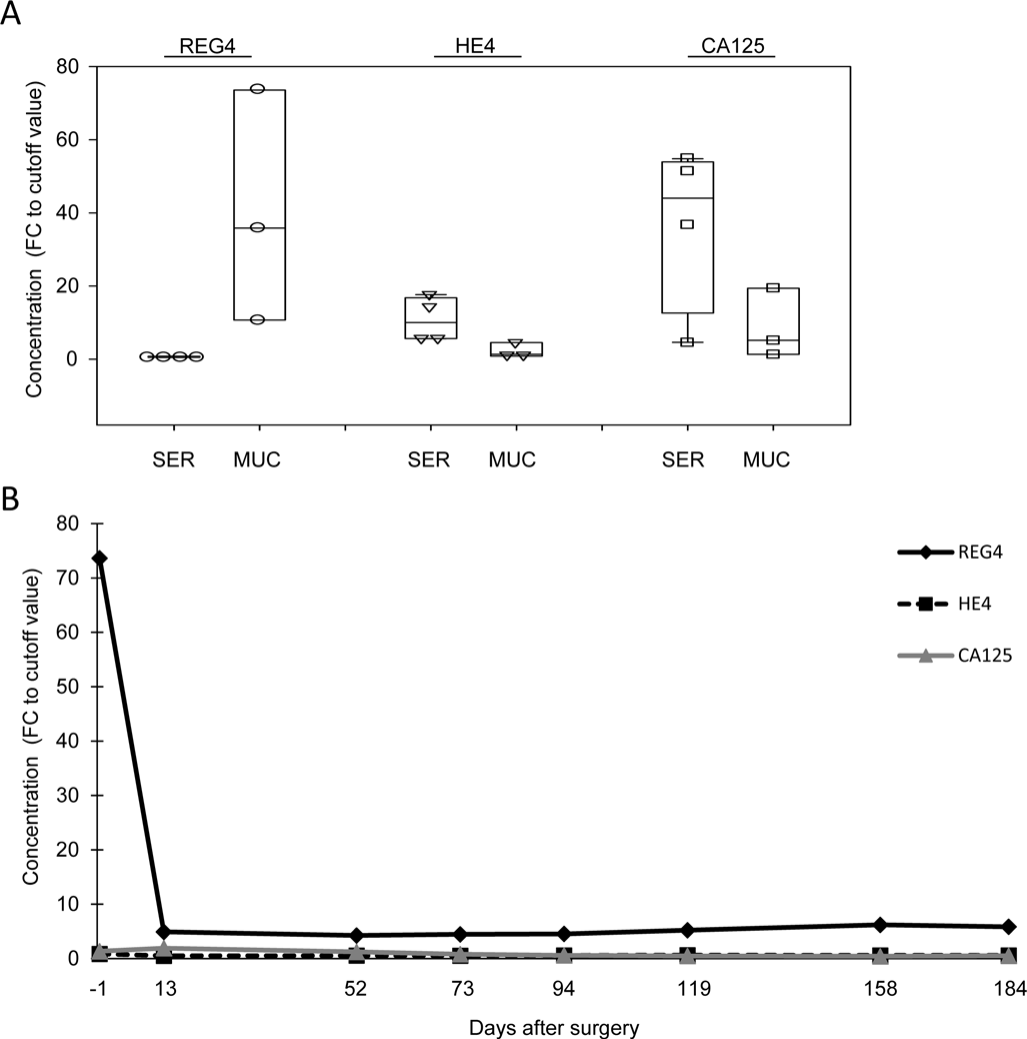
Secreted REG4 can be detected from serum and ascites of mucinous cancer patients. (A) ELISA analysis of REG4 protein concentration in sera of patients diagnosed with serous (SER) or mucinous (MUC) ovarian carcinomas. (B) REG4 protein concentrations in a series of pre- (day -1) and post-operative serum samples of one patient with diagnosed mucinous carcinoma.

To analyze the potential of REG4 as a serum biomarker for mucinous ovarian cancer, we used a REG4 specific sandwich ELISA assay. The samples included sera and ascites fluid from patients with serous or mucinous ovarian tumors. Detailed sample description and results are presented in S2 Table. The serum samples were taken before (pre-operative) and after (post-operative) surgery at several time points during follow-up. Ascites fluid was collected during surgery. To assess the specificity of REG4 for mucinous ovarian cancer histotype, we analyzed CA125, HE4 and REG4 concentrations from preoperative serum samples of ovarian cancer patients with high-grade serous (n = 4) or mucinous (n = 3) cancers. All preoperative sera were from patients with disseminated (stage III-IV) disease. The results are shown in Fig 4A. In order to be able to directly compare the ELISA-assays with different units of measure, the results are presented as fold-change (FC) of the respective malignancy indicating cut-off value (35 kU/l for CA125 and 150 pM for HE4). The chosen cut-off values are based on current recommendations. Numerical values are presented in S2 Table. For REG4 the cut-off value was set to 2 μg/l based on the highest REG4 concentration obtained from healthy male and a larger set of non-mucinous serum controls (S3 Table). A FC value > 1 is considered as a positive result suggestive for malignancy, *i*.*e*. higher marker concentration than normally present in healthy individuals. FC values ≤ 1 are considered as a negative results. The commonly used serum biomarkers CA125 and HE4 showed elevated concentrations in the sera of high-grade serous cancer patients (FC > 1) whereas the REG4 concentration remained below the cut-off value (FC < 1) (Fig 4A). In the sera of mucinous cancer patients, the concentration of CA125 and HE4 remained low. In these three samples the REG4 level was 10–70 fold over the cut-off value. Although the amount of samples was low, these results indicate that REG4 is specific for mucinous type tumors and can be detected at high concentrations in the sera of patients with disseminated mucinous cancer. In addition to serum samples, REG4 concentration was measured from cell-free ascites fluids of two patients with mucinous tumors (M1 and M2, S2 Table). Both samples were positive (33 pg/ml and 187 pg/ml, respectively) for REG4 protein indicating that mucinous tumor cells secrete REG4 also to the ascites fluid.

In order to determine the correlation of REG4, CA125 and HE4 concentrations to tumor burden, serum samples were measured at multiple time points from a patient (M1) treated for disseminated mucinous carcinoma (see S4 Table for detailed results). The results (FC of cut-off value, Fig 4B) showed that the high concentration of REG4 (FC = 73.6) detected in the preoperative sample (day 0) declined rapidly after surgery (day 13; FC = 4.9) and stayed on a low level albeit higher than the cut-off value 2 μg/l. The concentration of CA125 indicated malignancy in the three first time points (47 kU/l, 67 kU/l and 43 kU/l, respectively) after which it fell below the cut-off value of 35 kU/l, while the levels of HE4 remained under the cut-off value of 150 pM in all samples. This suggests that the REG4 concentration correlates to tumor burden better than CA125 or HE4 in mucinous ovarian cancer. While CA125 measurement indicated malignancy in the preoperative sample, HE4 measurement failed to detect the tumor in serum samples.

## Discussion

Ovarian neoplasms differ in their genetic profiles, biological behavior and outcome, and should be treated as distinct diseases already during surgical intervention. Thus, a specific preoperative diagnosis is crucial for selection of optimal treatment strategy. Currently there are no methods for preoperative discrimination of ovarian cancer subtypes and the specific diagnosis is reached only by perioperative frozen section or postoperative histopathological analysis. Here we suggest REG4 as a promising serum biomarker for separating mucinous cancers from other epithelial ovarian cancer subtypes. According to our results, serum REG4 analysis may also have potential for differentiation between benign and malignant mucinous neoplasms and for follow-up of mucinous ovarian cancer patients.

The specific mucinous lineage expression of REG4 within ovarian neoplasms was demonstrated by several ways. The first evidence was obtained from *in silico* analyses, which indicated very specific mRNA expression profile of REG4 in mucinous cancers among various ovarian tumor subtypes. REG4 expression was further validated with qRT-PCR and western blotting from fresh frozen ovarian tumor tissue samples originating from serous and mucinous subtypes. As expected based on the *in silico* data, REG4 was highly expressed only in malignant mucinous ovarian tumors. Finally, immunohistochemical staining of a tumor tissue microarray consisting of 185 tumor cores from benign and malignant serous, endometroid and mucinous neoplasms confirmed restricted expression in the mucinous lineage. Interestingly, no REG4 expression was detected in mucinous cystadenomas suggesting that immunohistochemical REG4 analysis could be used as an additional method for distinction between benign and malignant mucinous ovarian tumors. All the mucinous samples were confirmed to be of primary ovarian tumor origin. Our sample set did not contain specimens of the more rare types of mucinous ovarian carcinoma; such as endocervical/ seromucinous or foveolar types. Therefore, in order to determine the true potential of REG4 as an immunohistochemical marker, additional analyses with wider range of different types of samples are needed.

The serum biomarkers CA125 and HE4 are used for ovarian cancer diagnostics and follow-up. These markers mainly detect serous cancers [4,18,19], but are unreliable for diagnosis of non-serous histotypes, especially the mucinous subtype [20,21]. Here, we compared serum samples from patients with high-grade serous or mucinous ovarian cancer. Our results corroborate with earlier studies since in our sample set both CA125 and HE4 were significantly elevated in preoperative serum samples of serous carcinoma patients, but remained low in the mucinous carcinomas. In contrast, in all three mucinous cancer patients’ sera, REG4 level was elevated more than ten-fold over the highest value of the control sera. The serum REG4 level was not elevated in any of the serous cancer patients’ serum samples. Thus, a combination of REG4 with CA125 and HE4 could provide a more comprehensive marker panel for preoperative diagnosis of potentially neoplastic ovarian masses, and enable distinction of mucinous ovarian cancer from other ovarian cancer histotypes. However, as some malignant tumors did not show positive REG4 staining, we cannot exclude the possibility that a fraction of mucinous cancers could remain undetectable in serum REG4 analysis. Since our serum sample collection was limited, further analyses are needed to fully determine the role of REG4 serum measurement as a pre-operative method for mucinous ovarian cancer diagnosis.

REG4 has been previously indicated as a serum biomarker in gastric, colorectal and prostate cancer [22–24]. Thus, REG4 serum analysis alone may not be able to distinguish between primary mucinous ovarian cancer and ovarian metastases originating from the gastrointestinal tract. On the other hand, addition of REG4 analysis from serum on the side of CA125 and/or HE4 could provide a possibility for postoperative follow-up of mucinous ovarian cancer patients with a confirmed diagnosis. Indication for this came from a longitudinal series of serum samples from a patient with mucinous cancer. In this patient, the high preoperative serum REG4 concentration was reduced rapidly postoperatively and remained low during the uneventful follow-up period. To obtain definite proof of the utility of serum REG4 in disease control, however, more patients with longitudinal serum samples should be tested.

The function of REG4 in ovarian cancer is poorly understood and has not been widely investigated. A recent study suggests that REG4 modulates proliferation, apoptosis, migration and invasion of ovarian cancer cells, and has an important role in early ovarian carcinogenesis [25]. This study also reports immunohistochemical staining of REG4 from different ovarian cancer subtypes. Serum REG4 values were not quantified in any ovarian cancer subtypes. In contrast to our results, positive REG4 staining (from weak to strong) was also found in serous samples. In our material REG4 protein expression was either very high or very low, and no intermediate staining was observed. However, the qRT-PCR analysis of REG4 mRNA expression from fresh frozen mucinous ovarian tumors identified samples with intermediate or low expression, yet no expression was detected in samples from serous ovarian tumors. This might be due to differences in antibody specificity and the scaling of staining strength.

In conclusion, we identified REG4 as a potential biomarker for subtype specific diagnosis of mucinous ovarian cancer as well as for disease follow-up. According to our results REG4 protein is secreted into circulation by mucinous ovarian tumor cells and can be detected with an ELISA-based test. We measured three biomarkers from patient sera; CA125, HE4 and REG4 and found high concentrations of REG4 in preoperative samples of mucinous ovarian cancer patients, whereas CA125 and HE4 failed to convincingly detect malignancy in these samples. Our study suggests that combining REG4 with CA125 and HE4 could provide added value for preoperative diagnostics of ovarian masses of potential neoplastic origin, and enable distinction of mucinous ovarian cancer from other ovarian cancer subtypes with non-invasive methods.

## Supporting Information

S1 Table Genes with mucinous specific expression profile in clinical ovarian cancer samples. (XLSX)

S2 Table Detailed sample description and results of the ELISA assay. (DOCX)

S3 Table REG4 cut-off value has been set based on the highest REG4 concentration obtained from healthy male and a larger set of non-mucinous serum controls. (DOCX)

S4 Table Detailed results of serum ELISA-analysis at multiple time points from one patient. (DOCX)

S1 Fig *In Silico* analysis of *REG4* mRNA expression in the MediSapiens database across malignant human tissue samples (n = 15392). (PDF)

S2 Fig *In Silico* analysis of *REG4* mRNA expression in the MediSapiens database across healthy human tissue samples (n = 3082). (PDF)

## References

[1] Siegel R, Ma J, Zou Z, Jemal A. Cancer statistics, 2014. CA Cancer J Clin. 2014;64: 9–29. 10.3322/caac.21208 24399786

[2] Vaughan S, Coward JI, Bast RC Jr, Berchuck A, Berek JS, Brenton JD, et al Rethinking ovarian cancer: recommendations for improving outcomes. Nat Rev Cancer. 2011;11: 719–725. 10.1038/nrc3144 21941283 PMC3380637

[3] Coward JI, Middleton K, Murphy F. New perspectives on targeted therapy in ovarian cancer. Int J Womens Health. 2015;7: 189–203. 10.2147/IJWH.S52379 25678824 PMC4324539

[4] Moore RG, Brown AK, Miller MC, Skates S, Allard WJ, Verch T, et al The use of multiple novel tumor biomarkers for the detection of ovarian carcinoma in patients with a pelvic mass. Gynecol Oncol. 2008;108: 402–408. 18061248 10.1016/j.ygyno.2007.10.017

[5] Hess V, A'Hern R, Nasiri N, King DM, Blake PR, Barton DP, et al Mucinous epithelial ovarian cancer: a separate entity requiring specific treatment. J Clin Oncol. 2004;22: 1040–1044. 15020606 10.1200/JCO.2004.08.078

[6] Pectasides D, Fountzilas G, Aravantinos G, Kalofonos HP, Efstathiou E, Salamalekis E, et al Advanced stage mucinous epithelial ovarian cancer: the Hellenic Cooperative Oncology Group experience. Gynecol Oncol. 2005;97: 436–441. 15863142 10.1016/j.ygyno.2004.12.056

[7] Sung PL, Chang YH, Chao KC, Chuang CM, Task Force on Systematic Review and Meta-analysis of Ovarian Cancer. Global distribution pattern of histological subtypes of epithelial ovarian cancer: a database analysis and systematic review. Gynecol Oncol. 2014;133: 147–154. 10.1016/j.ygyno.2014.02.016 24556058

[8] Ludwick C, Gilks CB, Miller D, Yaziji H, Clement PB. Aggressive behavior of stage I ovarian mucinous tumors lacking extensive infiltrative invasion: a report of four cases and review of the literature. Int J Gynecol Pathol. 2005;24: 205–217. 15968194 10.1097/01.pgp.0000159935.38913.57

[9] Tabrizi AD, Kalloger SE, Kobel M, Cipollone J, Roskelley CD, Mehl E, et al Primary ovarian mucinous carcinoma of intestinal type: significance of pattern of invasion and immunohistochemical expression profile in a series of 31 cases. Int J Gynecol Pathol. 2010;29: 99–107. 10.1097/PGP.0b013e3181bbbcc1 20173494

[10] Prat J. Subclassification of ovarian cancer based on pathology and genetics. Eur J Cancer. 2009;45 Suppl 1: 427–428. 10.1016/S0959-8049(09)70076-0 19775658

[11] Kilpinen S, Autio R, Ojala K, Iljin K, Bucher E, Sara H, et al Systematic bioinformatic analysis of expression levels of 17,330 human genes across 9,783 samples from 175 types of healthy and pathological tissues. Genome Biol. 2008;9: R139-2008-9-9-r139. Epub 2008 Sep 19. 10.1186/gb-2008-9-9-r139 PMC2592717 18803840

[12] Myohanen TT, Pyykko E, Mannisto PT, Carpen O. Distribution of prolyl oligopeptidase in human peripheral tissues and in ovarian and colorectal tumors. J Histochem Cytochem. 2012;60: 706–715. 10.1369/0022155412453051 22740343 PMC3524555

[13] Heiskala K, Giles-Komar J, Heiskala M, Andersson LC. High expression of RELP (Reg IV) in neoplastic goblet cells of appendiceal mucinous cystadenoma and pseudomyxoma peritonei. Virchows Arch. 2006;448: 295–300. 16323007 10.1007/s00428-005-0105-1

[14] Kaprio T, Hagstrom J, Mustonen H, Koskensalo S, Andersson LC, Haglund C. REG4 independently predicts better prognosis in non-mucinous colorectal cancer. PLoS One. 2014;9: e109600 10.1371/journal.pone.0109600 25295732 PMC4190354

[15] Mpindi JP, Sara H, Haapa-Paananen S, Kilpinen S, Pisto T, Bucher E, et al GTI: a novel algorithm for identifying outlier gene expression profiles from integrated microarray datasets. PLoS One. 2011;6: e17259 10.1371/journal.pone.0017259 21365010 PMC3041823

[16] Halila H, Lehtovirta P, Stenman UH. Tumour-associated trypsin inhibitor (TATI) in ovarian cancer. Br J Cancer. 1988;57: 304–307. 3162682 10.1038/bjc.1988.67 PMC2246526

[17] Zhang YW, Ding LS, Lai MD. Reg gene family and human diseases. World J Gastroenterol. 2003;9: 2635–2641. 14669303 10.3748/wjg.v9.i12.2635 PMC4612022

[18] Holcomb K, Vucetic Z, Miller MC, Knapp RC. Human epididymis protein 4 offers superior specificity in the differentiation of benign and malignant adnexal masses in premenopausal women. Am J Obstet Gynecol. 2011;205: 358.e1–358.e6. 21722869 10.1016/j.ajog.2011.05.017

[19] Hellstrom I, Raycraft J, Hayden-Ledbetter M, Ledbetter JA, Schummer M, McIntosh M, et al The HE4 (WFDC2) protein is a biomarker for ovarian carcinoma. Cancer Res. 2003;63: 3695–3700. 12839961

[20] Miller K, Millar J, McCluggage WG. Emergence of CA125 immunoreactivity in recurrent or metastatic primary ovarian mucinous neoplasms of the intestinal type. Am J Surg Pathol. 2011;35: 1331–1336. 10.1097/PAS.0b013e3182233fce 21836488

[21] Kelly PJ, Archbold P, Price JH, Cardwell C, McCluggage WG. Serum CA19.9 levels are commonly elevated in primary ovarian mucinous tumours but cannot be used to predict the histological subtype. J Clin Pathol. 2010;63: 169–173. 10.1136/jcp.2009.072355 20154039

[22] Mitani Y, Oue N, Matsumura S, Yoshida K, Noguchi T, Ito M, et al Reg IV is a serum biomarker for gastric cancer patients and predicts response to 5-fluorouracil-based chemotherapy. Oncogene. 2007;26: 4383–4393. 17237819 10.1038/sj.onc.1210215

[23] Oue N, Kuniyasu H, Noguchi T, Sentani K, Ito M, Tanaka S, et al Serum concentration of Reg IV in patients with colorectal cancer: overexpression and high serum levels of Reg IV are associated with liver metastasis. Oncology. 2007;72: 371–380. 10.1159/000113147 18187959

[24] Hayashi T, Matsubara A, Ohara S, Mita K, Hasegawa Y, Usui T, et al Immunohistochemical analysis of Reg IV in urogenital organs: Frequent expression of Reg IV in prostate cancer and potential utility as serum tumor marker. Oncol Rep. 2009;21: 95–100. 19082448

[25] Chen S, Gou WF, Zhao S, Niu ZF, Zhao Y, Takano Y, et al The role of the REG4 gene and its encoding product in ovarian epithelial carcinoma. BMC Cancer. 2015;15: 471-015-1435-2. 10.1186/s12885-015-1435-2 PMC4469329 26077911

