# Supplementary material for: REG4 Is Highly Expressed in Mucinous Ovarian Cancer: A Potential Novel Serum Biomarker
Source: PLoS One. 2016 Mar 16;11(3):e0151590. doi: 10.1371/journal.pone.0151590 (PMC4794165; doi:10.1371/journal.pone.0151590)
Supplement: S2 Table — (DOCX) [file pone.0151590.s002.docx]

| **Supplementary Table S2.** Detailed description of samples and results of the serum ELISA-analysis. | | | | | | |  |  |  |
| --- | --- | --- | --- | --- | --- | --- | --- | --- | --- |
|  |  |  | |  |  | |  |  |  |
|  |  | Cut-off value 2 µg/l | | Cut-off value 150 pM | | | | Cut-off value 35 kU/l | |
| **Sample** | **Diagnosis** | **REG4 (µg/l)** | **FC** | **HE4 (pM)** | | **FC** | | **CA-125 (kU/l)** | **FC** |
| S1 | Serous carcinoma (Grade 3, stage IIIC/IV) | 1616 | 0.8 | 2643 | | 17.6 | | 1285 | 36.7 |
| S2 | Serous carcinoma (Grade 3, stage IIIC) | 1044 | 0.5 | 862 | | 5.7 | | 1918 | 54.8 |
| S3 | Serous carcinoma (Grade 3, stage IV) | 1274 | 0.6 | 2138 | | 14.3 | | 161 | 4.6 |
| S4 | Serous carcinoma (Grade 3, stage IIIC) | 938 | 0.5 | 837 | | 5.6 | | 1797 | 51.3 |
| M1 | Mucinous carcinoma (Grade 1, stage IV) | 147220 | 73.6 | 129 | | 0.9 | | 47 | 1.3 |
| M2 | Mucinous carcinoma | 71716 | 35.9 | 679 | | 4.5 | | 679 | 19.4 |
| M3 | Mucinous carcinoma (Grade 1, stage IC) | 21392 | 10.7 | 202 | | 1.3 | | 181 | 5.2 |
