# Supplementary material for: REG4 Is Highly Expressed in Mucinous Ovarian Cancer: A Potential Novel Serum Biomarker
Source: PLoS One. 2016 Mar 16;11(3):e0151590. doi: 10.1371/journal.pone.0151590 (PMC4794165; doi:10.1371/journal.pone.0151590)
Supplement: S3 Table — (DOCX) [file pone.0151590.s003.docx]

**Supplementary Table S3.** REG4 cut‑off value has been set based on the highest REG4 concentration obtained from healthy male and a larger set of non‑mucinous serum controls.

| **Sample code** | **REG4 (µg/l)** |
| --- | --- |
| SER_C_1 | 1,6 |
| SER_C_2 | 1,0 |
| SER_C_3 | 1,0 |
| SER_C_4 | 1,0 |
| SER_C_5 | 1,1 |
| SER_C_6 | 1,3 |
| SER_C_7 | 1,3 |
| SER_C_8 | 0,9 |
| SER_C_9 | 1,1 |
| SER_C_10 | 0,9 |
| SER_C_11 | 1,4 |
| SER_C_12 | 1,2 |
| SER_C_13 | 1,0 |
| SER_C_14 | 0,8 |
| SER_C_15 | 0,3 |
| SER_C_16 | 0,4 |
