# Supplementary material for: REG4 Is Highly Expressed in Mucinous Ovarian Cancer: A Potential Novel Serum Biomarker
Source: PLoS One. 2016 Mar 16;11(3):e0151590. doi: 10.1371/journal.pone.0151590 (PMC4794165; doi:10.1371/journal.pone.0151590)
Supplement: S4 Table — (DOCX) [file pone.0151590.s004.docx]

| **Supplementary Table S4.** Detailed results of serum ELISA-analysis at multiple time points from one patient. | | | | | | | | | |  |  |
| --- | --- | --- | --- | --- | --- | --- | --- | --- | --- | --- | --- |
|  |  | | |  |  | |  |  | | |  |
|  | Cut-off value 2 µg/l | | Cut-off value 150 pM | | | | Cut-off value 35 kU/l | | |  |  |
| **Day (p.o.)** | **REG4 (µg/l)** | **FC** | **HE4 (pM)** | | | **FC** | **CA-125 (kU/l)** | | **FC** |  |  |
| -1 | 147 | 73,6 | 129 | | | 0,9 | 47 | | 1,3 |  |  |
| 13 | 10 | 4,9 | 78 | | | 0,5 | 67 | | 1,9 |  |  |
| 52 | 8 | 4,2 | 84 | | | 0,6 | 43 | | 1,2 |  |  |
| 73 | 9 | 4,5 | 82 | | | 0,5 | 28 | | 0,8 |  |  |
| 94 | 9 | 4,5 | 89 | | | 0,6 | 21 | | 0,6 |  |  |
| 119 | 10 | 5,2 | 95 | | | 0,6 | 17 | | 0,5 |  |  |
| 158 | 12 | 6,2 | 88 | | | 0,6 | 15 | | 0,4 |  |  |
| 184 | 12 | 5,8 | 88 | | | 0,6 | 18 | | 0,5 |  |  |
