# Supplementary figures and images for: REG4 Is Highly Expressed in Mucinous Ovarian Cancer: A Potential Novel Serum Biomarker
Source: PLoS One. 2016 Mar 16;11(3):e0151590. doi: 10.1371/journal.pone.0151590 (PMC4794165; doi:10.1371/journal.pone.0151590)

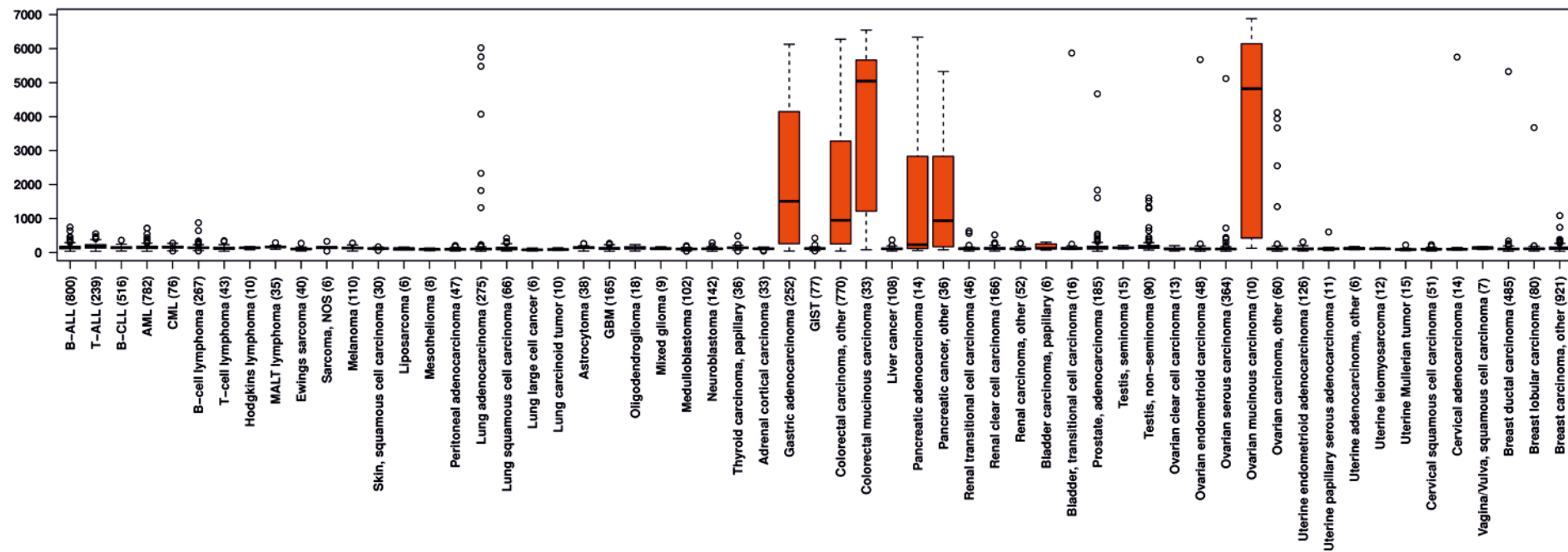

Supplement: S1 Fig — (PDF) [file pone.0151590.s005.pdf]

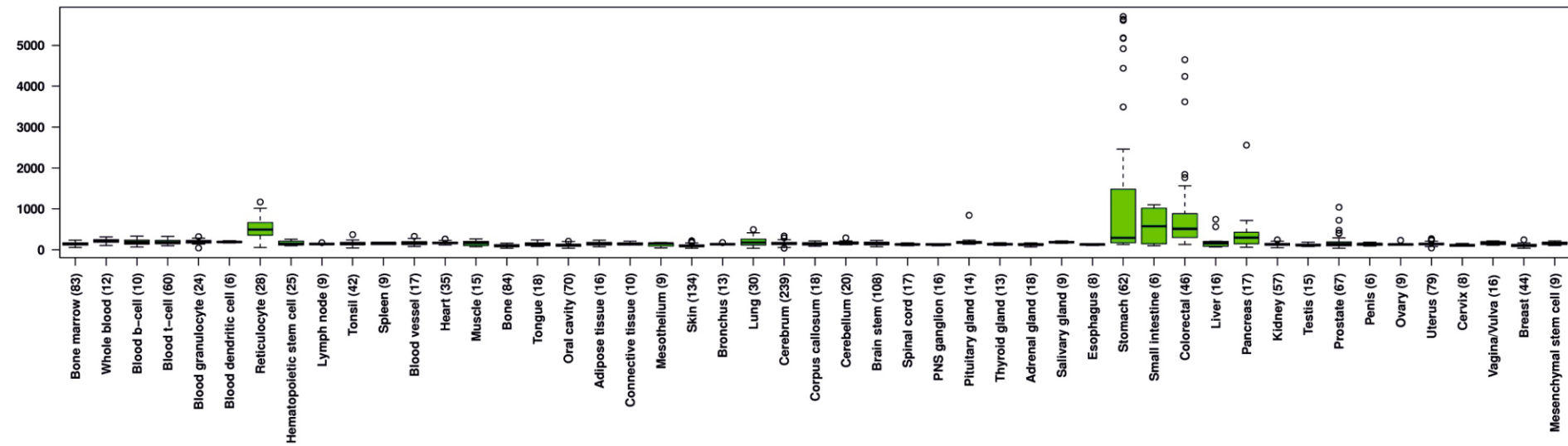

Supplement: S2 Fig — (PDF) [file pone.0151590.s006.pdf]
